# Effect of Lipopolysaccharides on Liver Tumor Metastasis of *twist1a/kras^V12^* Double Transgenic Zebrafish

**DOI:** 10.3390/biomedicines10010095

**Published:** 2022-01-02

**Authors:** Jeng-Wei Lu, Liang-In Lin, Yuxi Sun, Dong Liu, Zhiyuan Gong

**Affiliations:** 1 Department of Biological Sciences, National University of Singapore, Singapore 117543, Singapore; e0437708@u.nus.edu; 2 Department of Clinical Laboratory Sciences and Medical Biotechnology, National Taiwan University, Taipei 10048, Taiwan; lilin@ntu.edu.tw; 3 Department of Laboratory Medicine, National Taiwan University Hospital, Taipei 10048, Taiwan; 4 Brain Research Center, School of Life Sciences, Southern University of Science and Technology, Shenzhen 518055, China; liud@sustech.edu.cn

**Keywords:** liver tumor metastasis, lipopolysaccharides, *twist1a*, *kras^V12^*, transgenic zebrafish

## Abstract

The poor prognosis of patients diagnosed with hepatocellular carcinoma (HCC) is directly associated with the multi-step process of tumor metastasis. *TWIST1*, a basic helix-loop-helix (bHLH) transcription factor, is the most important epithelial-mesenchymal transition (EMT) gene involved in embryonic development, tumor progression, and metastasis. However, the role that *TWIST1* gene plays in the process of liver tumor metastasis in vivo is still not well understood. Zebrafish can serve as a powerful model for cancer research. Thus, in this study, we crossed *twist1a*+ and *kras*+ transgenic zebrafish, which, respectively, express hepatocyte-specific mCherry and enhanced green fluorescent protein (EGFP); they also drive overexpression of their respective transcription factors. This was found to exacerbate the development of metastatic HCC. Fluorescence of mCherry and EGFP-labeled hepatocytes revealed that approximately 37.5% to 45.5% of the *twist1a*+/*kras*+ double transgenic zebrafish exhibited spontaneous tumor metastasis from the liver to the abdomen and tail areas, respectively. We also investigated the inflammatory effects of lipopolysaccharides (LPS) on the hepatocyte-specific co-expression of *twist1a*+ and *kras*+ in double transgenic zebrafish. Following LPS exposure, co-expression of *twist1a*+ and *kras*+ was found to increase tumor metastasis by 57.8%, likely due to crosstalk with the EMT pathway. Our results confirm that *twist1a* and *kras* are important mediators in the development of metastatic HCC. Taken together, our in-vivo model demonstrated that co-expression of *twist1a*+/*kras*+ in conjunction with exposure to LPS enhanced metastatic HCC offers a useful platform for the study of tumor initiation and metastasis in liver cancer.

## 1. Introduction

Hepatocellular carcinoma (HCC) is the fifth most common cancer in the world and a major threat to human health [1,2,3]. Despite substantial progress in the treatment of HCC in recent years, 600,000 people still die from this disease annually, making it the third leading cause of cancer-related deaths worldwide [4,5]. Liver resection and transplantation are the most common HCC treatment methods; however, high recurrence and metastasis rates still lead to poor prognosis in HCC patients [6]. Therefore, early diagnosis and treatment of HCC are critical. A small number of genes have been linked to the occurrence and metastasis of HCC; however, elucidating the mechanisms which underlie liver tumor metastasis remains a pressing issue [7].

The significance of epithelial-mesenchymal transition (EMT) in the process of cancer metastasis has been explored previously. A large number of studies have found that EMT plays a key role in tumor invasion and metastasis, and *TWIST1* has been identified as an important regulating factor in the EMT process. *TWIST1* is a basic helix-loop-helix (bHLH) transcription factor and is also one of the most important EMT genes involved in embryonic development, tumor progression, and metastasis [8,9]. The fact that EMT has been shown to regulate various biological processes in tumors, including drug resistance, demonstrates its complexity [10]. *TWIST1* negatively regulates *E-cadherin* and positively regulates *Vimentin*. Both of these proteins are important to EMT induction. Dysregulation of *TWIST1* expression is associated with the *E-cadherin*-mediated loss of intercellular adhesion, activation of mesenchymal markers, and induction of cell motility [11]. Nonetheless, many aspects of EMT remain unclear and require further study. In particular, research is required to identify and understand EMT-related genes.

Abnormal expression of *TWIST1* has been frequently observed in many types of cancers. The upregulation of *TWIST1* in HCC cell lines promotes the proliferation, cell migration, invasion, and metastasis of cancer cells [7,12,13,14]. Overexpression of *TWIST1* is also associated with shorter overall survival in HCC patients [15]. Research into *TWIST1* has broad applications and potential therapeutic value for HCC. However, the relationship between *TWIST1* and the proto-oncogene *K-RAS*, which is a member of the RAS protein family and is mutated in a high percentage of human liver cancers, in HCC is unclear.

RAS proteins are a family of small molecular switches regulated by guanosine triphosphate, which can transmit signals from the cell membrane to the nucleus and activate a variety of signaling pathways involved in cell proliferation, transformation, and tumor progression. RAS family proteins include H-RAS, N-RAS, and K-RAS [16,17,18]. Many single-point mutations in *RAS* genes result in the constitutive activation of *RAS* with impaired GTPase activity, which leads to continuous stimulation of cell proliferation. The frequency of these gene mutations varies in different tumor types. In total, approximately 30% of human tumors have *RAS* gene mutations, and these mutations most commonly occur in the *K-RAS* gene [17]. For example, *K-RAS* mutations have been identified in 77% of human liver cancers, which is higher than the incidence of mutations in *H-RAS* and *N-RAS* in these cancers. The activation of RAS protein signals, which leads to the proliferation and transformation of hepatocytes, has also been observed in human HCC specimens [16,18,19].

Many microenvironmental inflammatory factors have been identified as potential therapeutic targets for HCC [20]. Aspirin, a non-steroidal anti-inflammatory drug, has been shown to reduce the risk of HCC and improve survival [21]. In addition, upregulation of Toll-like receptor (TLR) signaling, which is associated with inflammation-related cancers, has been found to play a key role in the prognosis of chronic and inflammatory diseases that lead to HCC [22]. Lipopolysaccharides (LPS), which are large molecules composed of lipids and polysaccharides that exist in the outer membrane of gram-negative bacteria, function by binding to toll-like receptor 4 (TLR4). In HCC, the cooperation of TLR4 and toll-like receptor 9 (TLR9) may activate the signal transducer and activator of transcription 3 (STAT3) [23,24,25,26]. Exposure to LPS leads to tumor growth and angiogenesis in HCC via the TLR4 receptor in vivo. The signaling which occurs following induction by LPS also promotes EMT in HCC [25,27,28].

The occurrence of tumors can be clearly divided into three independent stages: tumor initiation, progression, and metastasis [29,30]. In previous research, we characterized novel roles of *twist1a* and *xmrk* (an activated epidermal growth factor receptor (EGFR) homolog) in tumorigenesis and metastasis and proposed a new animal model for screening anti-tumor metastasis drugs [31,32,33]. However, no reports pertaining to the use of animal models in the study of how *TWIST1* and *K-RAS* affect the initiation and maintenance of liver tumorigenesis have been published.

The use of zebrafish in the study of liver disease and HCC has recently become more widespread [34]. Thus, in the current study, we first investigated the potential relevance of *twist1a* and *kras* in liver tumors using a zebrafish model. We also explored the in-vivo mechanism which underlies the effects of LPS on liver tumors in *kras* or *twist1a*/*kras* transgenic zebrafish. Specifically, we were interested in how LPS promotes tumor progression and metastasis in these zebrafish. Results of this study helped to elucidate a new molecular mechanism of HCC and provided new insights pertaining to potential therapeutic targets against HCC.

## 2. Materials and Methods

### 2.1. Zebrafish Husbandry and Maintenance

All experimental protocols and procedures involving zebrafish were approved by the Institutional Animal Care and Use Committee (IACUC) of the National University of Singapore and National Taiwan University. These experiments were also conducted in accordance with the “Guide for the Care and Use of Laboratory Animals” of the National Institutes of Health. All zebrafish embryos and larvae were maintained in E3 medium. Adult zebrafish were maintained in a recirculating aquatic system at 28 °C with a 14-h light/10-h dark cycle in accordance with standard practice. Dry pellets (GEMMA Micro 150 and 300, Skretting Nutreco, Tooele, UT, USA) were fed to adult zebrafish twice a day at a designated amount of approximately 3% body mass and directly proportional to the density of zebrafish within the tank. The zebrafish were fed lab-grown brine shrimp or commercial fish feed three times per day [35,36].

### 2.2. Generation of fabp10a:twist1a/kras Double Transgenic Zebrafish

In brief, Both of fabp10a:mCherry-T2A-twist1a-ER^T2^ (abbreviated as *twist1a*+) and fabp10a:rtTA2s-M2;TRE2:EGFP-kras^V12^ (abbreviated as *kra*s+) transgenic zebrafish lines were generated in our previous work [31,37]. (These transgenic lines respectively expressed hepatocyte-specific *twist1*a and *kras^V12^*). The wild-type AB zebrafish strain was used as a control. To establish a fabp10a:twist1a/kras^V12^ double transgenic zebrafish (abbreviated as *twist1a*+/*kras*+), we crossed *twist1a+* and *kras+* transgenic zebrafish and then selected positive F1 larvae, which were maintained under the zebrafish husbandry conditions described above until reaching the adult stage and undergoing further research.

### 2.3. RNA Isolation and Reverse Transcription PCR (RT-PCR)

Total RNA was extracted from primary liver tumors, metastatic liver tumors, and adjacent normal tissue of adult zebrafish using the RNeasy Mini Kit (Qiagen, Hilden, Germany); 1 μg RNA was then reverse transcribed into complementary DNA (cDNA) using the QuantiTect Whole Transcriptome Kit (Qiagen, Hilden, Germany). We amplified cDNA templates via polymerase chain reaction (PCR) using exTEN 2× PCR Master Mix (Axil Scientific, Singapore, Singapore). To assess liver markers, expression of *fabp10a* (Primers: forward, CCAGTGACAGAAATCCAGCA; reverse, GTTCTGCAGACCAGCTTTCC), *tfa* (Primers: forward, TGCAGAAAAAGCTGGTGATG; reverse, ACAGCATGAACTGGCACTTG), and *actin* (Primers: forward, CTCCATCATGAAGTGCGACGT; reverse, CAGACGGAGTATTTGCGCTCA) internal control in adult primary liver tumors, metastatic liver tumors, and adjacent normal tissue, we employed RT-PCR according to the following protocol: 1 μL of cDNA was amplified for 1 cycle at 95 °C for 5 min; followed by 35 cycles at 95 °C for 10 s, 58 °C for 30 s, and 68 °C for 1 min. The cDNA was then incubated at 68 °C for an additional 7 min to allow for synthesis completion. The resulting PCR products were subjected to 1.0% agarose gel electrophoresis, in which actin was used as the internal control for the cDNA assay, in accordance with published primers and protocols [33,38,39].

### 2.4. Induction of Transgene Expression Using Doxycycline and 4-Hydroxytamoxifen Treatment

At 5 days post-fertilization (dpf), larvae were screened for positive mCherry and/or EGFP fluorescence (to identify *twist1a+* and/or *kras+* transgenic zebrafish, respectively) using a fluorescence stereo microscope (SMZ18, Nikon, Tokyo, Japan). Doxycycline (Dox, Sigma-Aldrich, St. Louis, MO, USA) and 4-Hydroxytamoxifen (4-OHT, Sigma-Aldrich, St. Louis, MO, USA) were used to respectively induce *kras* and *twis1ta* expression. Induction studies involving 3- to 5-month post-fertilization (mpf) adult fish were performed in a 5-L tank that contained fresh water (changed every other day). To maintain tumor growth and induce metastasis over the long-term, *twist1a*+, *kras*+, and *twist1a*+/*kras*+ transgenic zebrafish, as well as their wild-type siblings, were treated with 20 μg/mL Dox and 1 μg/mL 4-OHT for 2 and 4 weeks.

### 2.5. Induction of Transgene Expression and LPS Exposure in Transgenic Zebrafish

Each treatment group included 20 larvae that were incubated in 1× E3 medium and treated with 20 μg/mL Dox alone or with 20 μg/mL Dox + 40 ng/mL LPS (catalog number: L4391; Sigma-Aldrich, St. Louis, MO, USA) and then maintained in 6-well plates for 3 days. Adult zebrafish were treated with 10 μg/mL Dox alone or with 10 μg/mL Dox + 40 ng/mL LPS for 2 weeks. Following that, the double expression of *twist1a+/kras+* in transgenic zebrafish was induced via treatment with 10 μg/mL Dox and 1 μg/mL 4-OHT in 5-L tanks. The *twist1a+/kras+* treatment group was also exposed to 40 ng/mL LPS for 4 weeks. For these experiments, fresh water, Dox, 4-OHT, and LPS were changed every other day. The mortality of adult zebrafish was determined daily, and samples were collected to study long-term treatment effects.

### 2.6. Collection of Tissue, Paraffin Sectioning, and Histochemical Analysis

In accordance with published protocols [33,36,40], all zebrafish samples were collected following euthanization at 2- or 4-weeks post-induction (wpi). Liver tissues were then fixed and embedded in paraffin for histological analysis. Specifically, 5-μm sections were deparaffined, rehydrated, and examined using the EnVision™+ Dual Link System (Dako, Carpinteria, CA, USA) according to previous methodologies for immunohistochemistry (IHC) analysis. Primary antibodies included rabbit anti-PCNA (Dilution: 1:500; Catalog Number: FL-261, Santa Cruz, CA, USA), rabbit anti-Caspase-3 (Dilution: 1:200; Catalog Number: C92-065, BD Biosciences, San Diego, CA, USA), mouse anti-E-cadherin (Dilution: 1:200; Catalog Number: 610188, BD Biosciences, San Diego, CA, USA), and mouse anti-Vimentin (Dilution: 1:200; Catalog Number: 610188, Abcam, Cambridge, MA, USA), which were used to stain hepatic tissues of zebrafish at 4 °C overnight. After washing with 1× phosphate-buffered saline (PBS), peroxidase activity was detected by incubating tissue sections at room temperature with a universal secondary biotinylated antibody for 30 min and then adding Dako diaminobenzidine (DAB) substrate for development. Tissue sections were counterstained with Mayer’s hematoxylin before being dehydrated, cleared, and mounted with slide covers. An Axio Imager Z2 microscope (Zeiss LSM 880, Goettingen, Germany) was used to visualize the sections. Images were analyzed with constant acquisition setting (microscope, magnification, light intensity, exposure time) using a 200× or 400× microscope objective. The results of histochemical analysis and larval measurement were evaluated by two independent senior scientists or pathologists in a single-blind manner to evaluate.

### 2.7. Statistical Analysis

All statistical analyses were performed using GraphPad Prism 9 software (GraphPad Software, La Jolla, CA, USA), as previously described [36]. For all in-vivo experiments, a two-tailed unpaired Student’s t-test or one-way analysis of variance (ANOVA) was applied to compare experimental and control groups. To determine the overall survival of zebrafish, survival rates were derived using the Kaplan-Meier method and log-rank test. Quantification of the IHC data using Image J software (NIH, Bethesda, MD, USA). Significance was set at a *p*-value of 0.05 or less.

## 3. Results

### 3.1. Phenotype of twist1a+/kras+ Double Transgenic Zebrafish and Liver Tumor Metastasis Induced by Dox Treatment

For induction studies, 3 to 5 months of mpf adult zebrafish were used for treatment experiments. To comprehensively elucidate tumor progression and metastatic development in *twist1a*+/*kras*+ double transgenic zebrafish, experiments in this study employed *twist1a*+, *kras*+, and *twist1a*+/*kras*+ transgenic zebrafish, as well as their non-transgenic wild-type siblings. All zebrafish were treated with Dox and 4-OHT. Long-term treatment samples were collected and investigated at 2 and 4 weeks (Supplementary Figure S1A).

**Figure 1:**
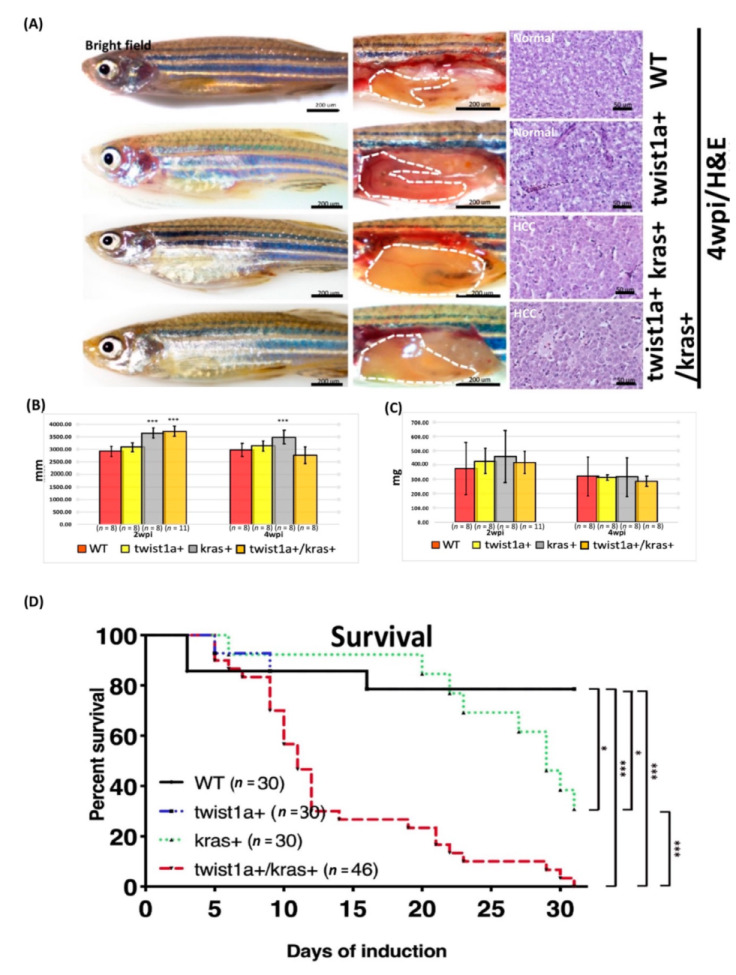

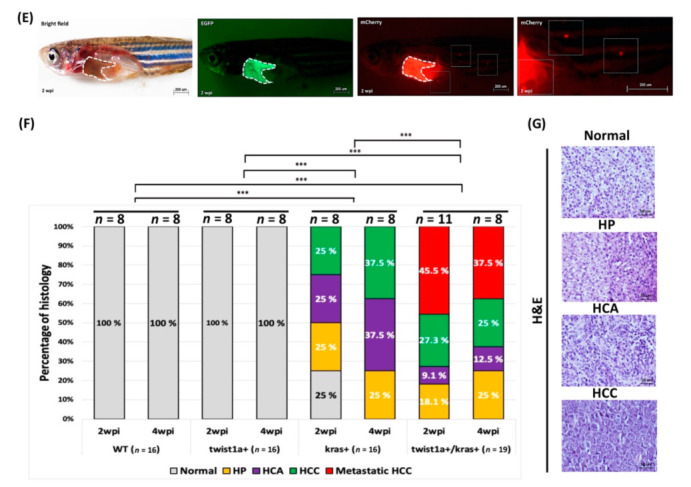
Induction of tumor metastasis in *twist1a+/kras+* transgenic zebrafish with Dox treatment. All zebrafish were treated with 20μg/mL Dox and 1μg/mL 4-OHT at 4 mpf, and samples were taken at 2 and 4 wpi. (**A**) Representative images of *wild-type*, *twist1a+*, *kras+*, and *twist1a+*/*kras+* transgenic zebrafish. The left column shows the external appearance, the middle column shows the internal organs of the abdomen with the liver outlined (white dotted line), and the right column shows H&E staining of liver tissues at 4 wpi. Compared with the wild-type group, (**B**) the body lengths of *kras+* and/or *twist1a+*/*kras+* transgenic zebrafish differed significantly at 2 and 4 wpi, whereas (**C**) the body weights of transgenic zebrafish did not differ at 2 and 4 wpi. (**D**) Kaplan-Meier survival curves showing the percentage of survival at 4 wpi. (**E**) Fluorescence analysis presenting evidence of metastatic HCC at 2 wpi in *twist1a*+/*kras*+ zebrafish (white dotted line: primary and metastatic liver tumors). (**F**) Histological analysis revealed that wild-type, *twist1a+*, *kras+*, and *twist1a+*/*kras+* transgenic zebrafish developed HCC or metastatic HCC at 4 wpi. (**G**) Representative images of normal, HP, HCA, and HCC using histological analysis. Scale bars: 50 or 200 μm. Student’s *t*-test or one-way ANOVA were used to assess differences between variables: * *p* < 0.05, *** *p* < 0.001.

To study how *twist1a+* and *kras+* affected tumor growth and liver tumor metastasis over the long term, all zebrafish groups (*twist1a+*, *kras+*, *twist1a+/kras+*, and wild-type) were treated with 20 μg/mL Dox and 1μg/mL 4-OHT for 4 weeks. Compared with wild-type and *twist1a+* control groups, an enlarged abdomen and obvious liver overgrowth were observed in both *kras+* and *twist1a*+/*kras*+ zebrafish at 2 and 4 wpi. Hematoxylin and eosin (H&E) staining revealed that all liver tumors in the *kras+* or *twist1a*+/*kras*+ zebrafish ranged from normal liver morphology to HCC and included the following classes: normal, hyperplasia (HP), hepatocellular adenoma (HCA), and HCC (Figure 1A,G). Zebrafish hepatocellular neoplasms have similar histological characteristics to human hepatocellular neoplasms during the growth process. Therefore, the classification of zebrafish liver neoplasm types was based on the criteria for rodent hepatocellular neoplasms and established criteria as previously studied [41,42,43,44]. The classification criteria for liver neoplasm types are as follows: (1) The normal liver has a typical two-cell hepatic plate structure, uniform shape, size and clear boundaries of the cell. (2) The hyperplasia maintains hepatic plate arrangement with an increased prominent nucleus. (3) Unclear hepatic plates with clear cell boundary and relatively uniformed cell shape were found at HCA. (4) HCC was characterized by loss of cell boundaries and hepatic plate structure, increased mitotic cells and multiple nucleus. Furthermore, the body lengths of *kras+* and *twist1a*+/*kras*+ transgenic zebrafish were significantly larger than those of the wild-type control; however, there was no significant difference in body weights among all groups (Figure 1B,C). A significantly higher mortality was observed in *kras+* or *twist1a*+/*kras*+ zebrafish (Figure 1D). The representative images and percentages of *kras+* and *twist1a*+/*kras*+ zebrafish exhibiting HCA or HCC development were as follows: HCA (2 wpi: 2/8; 1/11, respectively; 4 wpi: 3/8; 1/8, respectively) or HCC (2 wpi: 2/8; 3/11, respectively; 4 wpi: 3/8; 2/8, respectively). Some *twist1a*+/*kras*+ zebrafish also showed evidence of metastatic HCC at 2 and 4 wpi (2 wpi: 5/11; 4 wpi: 3/8) (Figure 1E–G).

### 3.2. Detection of fabp10a and tfa Expression in Primary and Metastatic Liver Tumors Tissues from twist1a+/kras+ Double Transgenic Zebrafish

**Figure 2:**
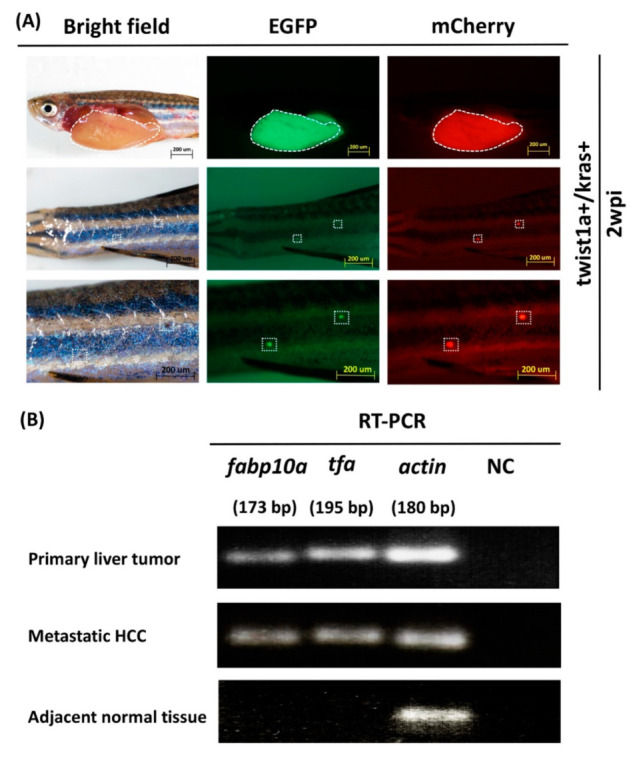
Liver markers in primary and metastatic liver tumor tissues from *twist1a+/kras+* double transgenic zebrafish. (**A**) Immunofluorescence was used to visualize mCherry or EGFP-labeled metastatic liver tumors in *twist1a*+/*kras*+ zebrafish (white dotted line: primary and metastatic liver tumors). (**B**) Semi-quantitative RT-PCR data showing expression levels of *fabp10a* and *tfa* in primary liver tumors, metastatic HCC tissues, and adjacent normal muscle tissues. *Actin* and non-template samples were respectively used as positive and negative controls. Scale bar: 200 μm.

To determine the expression of *fabp10a* and *tfa* at metastatic tumor cells, we collected primary liver tumors, metastatic HCC tissues, and adjacent normal muscle tissues on zebrafish body from *twist1a+*/*kras+* transgenic zebrafish following treatment with 20 μg/mL Dox and 1 μg/mL 4-OHT. At 2 wpi, EGFP and mCherry fluorescence signal of *twist1a+*/*kras+* zebrafish revealed evidence of metastatic HCC (Figure 2A). To identify the expression of *fabp10a* and *tfa* at tumors, we semi-quantified the mRNA expression of two zebrafish liver markers (*fabp10a* and *tfa*) in primary liver tumors tissues, metastatic HCC tissues, and adjacent normal muscle tissues using semi-quantitative RT-PCR. We found that *fabp10a* and *tfa* genes were expressed in both primary tumors and metastatic HCC tissues, confirming that metastatic HCC may come from the liver. Note that mRNA expression of *fabp10a* and *tfa* was not observed in adjacent normal muscle tissues. *Actin* and non-template samples respectively served as positive and negative controls (Figure 2B).

### 3.3. Co-Expression of twist1a/kras Significantly Increased Apoptosis, and twist1a Activated the EMT Pathway through E-Cadherin and Vimentin

**Figure 3:**
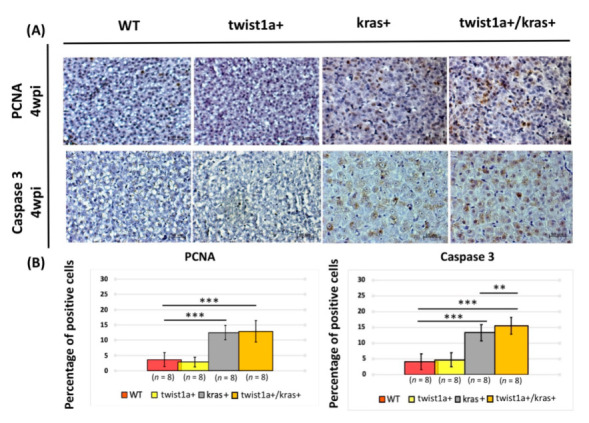
Main hallmarks of cell proliferation and cell apoptosis in liver tissues from *twist1a+/kras+* double transgenic zebrafish. (**A**) Immunohistochemical staining micrograph showing proliferating and apoptotic cells in liver cross-sections from wild-type, *twist1a*+, *kras*+, and *twist1a+/kras* zebrafish. (**B**) Quantification of the percentage of positive cells for the all fields following PCNA and caspase-3 staining at 4 wpi using ImageJ. Scale bar: 50 μm. Student’s *t*-tests were used to assess differences between variables: ** *p* < 0.01, *** *p* < 0.001.

To further compare the severity of liver tumorigenesis and metastasis between *kras*+ and *twist1a*+/*kras*+ zebrafish, the main hallmarks of cell proliferation and cell apoptosis, i.e., PCNA and caspase-3 staining, were examined in fish livers. After induction with 20 μg/mL Dox and 1 μg/mL 4-OHT of transgenic gene expression, *kras*+ and *twist1a*+/*kras*+ zebrafish showed a significant increase in the proliferation of liver cells and cell apoptosis compared with WT control zebrafish (Figure 3). At 4 wpi, we also found a dramatic increase in the percentage of *twist1a*+/*kras*+ zebrafish undergoing liver cell apoptosis compared with *kras*+ zebrafish (Figure 3A,B). Consistent with histological observations, the percentage of proliferating liver cells increased more rapidly in *kras*+ zebrafish and *twist1a*+/*kras*+ than in wild-type and *twist1a*+ control zebrafish from 2 and 4 wpi (Figure 1F); however, at 4 wpi, the percentage of liver cells undergoing apoptosis was greater in *twist1a*+/*kras*+ zebrafish than in *kras*+ zebrafish (Figure 3A,B). These observations were consistent with findings from our previous studies [32,36,45].

**Figure 4:**
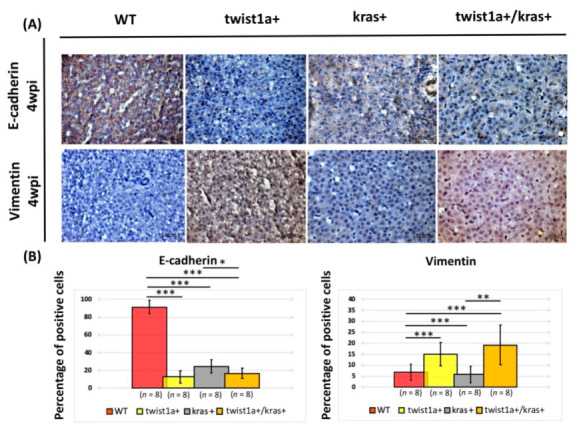
Main EMT hallmarks E-cadherin and Vimentin in liver tissues from *twist1a+/kras+* double transgenic zebrafish. (**A**) Immunohistochemical staining micrograph showing E-cadherin and Vimentin-positive cells in liver cross-sections of wild-type, *twist1a*+, *kras*+, and *twist1a+/kras+* zebrafish. (**B**) Quantification of the percentage of E-cadherin and Vimentin-positive cells for all fields following staining at 4 wpi using ImageJ. Scale bar: 50 μm. Student’s *t*-tests were used to assess differences between variables: * *p* < 0.05, ** *p* < 0.01, *** *p* < 0.001.

In order to identify a potential mechanism and pathway involved in liver tumorigenesis or metastasis, we further compared the severity of liver tumor occurrence and metastasis between *kras*+ and *twist1a*+/*kras*+ zebrafish. For this, the main EMT hallmarks during cancer metastasis, E-cadherin and Vimentin, were examined by immunohistochemical staining of the liver. Immunohistochemical staining results revealed that, following induction with 20 μg/mL Dox and 1 μg/mL 4-OHT, *kras*+ zebrafish liver tissue had a decrease in E-cadherin and a corresponding increase in Vimentin at 4 wpi compared with wild-type zebrafish. However, the *kras*+ zebrafish liver tissue also showed an increase in E-cadherin and corresponding decrease in Vimentin compared with *twist1a*+/*kras*+ zebrafish (Figure 4A). Quantification of the percentage of positive cells for E-cadherin and Vimentin supported these findings (Figure 4B). These observations suggest that co-expression of *twist1a*+ and *kras*+ could trigger crosstalk along the EMT pathway and could contribute to the liver tumorigenesis or metastasis in *kras*+ and *twist1a*+/*kras*+ zebrafish. These observations are also consistent with findings from our previous studies [33,40].

### 3.4. Exposure to LPS Increased Liver Size in kras+ Transgenic Zebrafish Larvae

**Figure 5:**
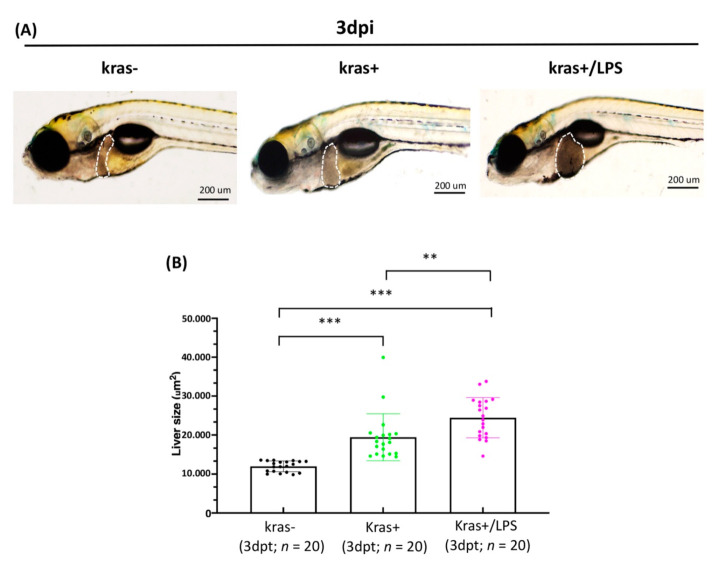
LPS increased liver size in *kras*+ transgenic zebrafish larvae. (**A**) Representative images and (**B**) quantification of liver size in wild-type (*kras*−) control, *kras*+, and *kras+*/LPS zebrafish at 3 dpi (white dotted frame: liver; black dots; green dots; pink dots: the number of zebrafish larvae, respectively). Scale bar: 200 μm. Student’s *t*-tests were used to assess differences between variables: ** *p* < 0.01, *** *p* < 0.001.

Before examining the long-term effects of LPS treatment, we examined the short-term effects of LPS treatment using *kras*+ transgenic larvae and non-transgenic wild-type sibling larvae. For this, four-day-old *kras*+ transgenic zebrafish larvae were treated with 20 μg/mL Dox alone or with 20 μg/mL Dox + 40 ng/mL LPS for 3 days. Wild-type (*kras*−) zebrafish larvae treated with 20 μg/mL Dox without exposure to LPS served as controls. All larvae in each group were imaged, and sizes of the livers were quantified (Figure 5A). Exposure to LPS significantly increases liver size in *kras*+/LPS larvae, compared with *kras*+ and *kras*− control larvae (Figure 5B).

### 3.5. Liver Tumor Phenotypes Induced by Sustained Expression of kras and Exposure to LPS in Adult Transgenic Zebrafish

**Figure 6:**
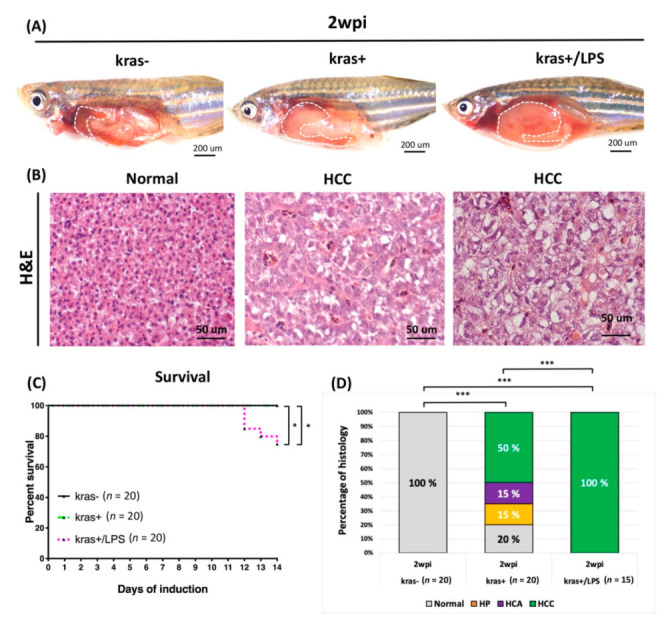
LPS promoted HCC progression in adult *kras*+ transgenic zebrafish. The wild-type (*kras*−) control, *kras+,* and *kras+*/*LPS* transgenic zebrafish were treated at 4 mpf with 10μg/mL Dox alone or with 10μg/mL Dox + 40ng/mL LPS, and samples were taken at 2 wpi. (**A**) The upper row shows the internal organs of the abdomen, and (**B**) the lower row displays H&E staining of liver sections (white dotted frame: liver). (**C**) Kaplan-Meier survival curves reveal the percentage of survival at 2 wpi. (**D**) Histological analysis shows that *kras+* and *kras+*/*LPS* transgenic zebrafish developed HCC at 2 wpi. Scale bar: 50 or 200 μm. Student’s *t*-test or one-way ANOVA were used to assess differences between variables: * *p* < 0.05, *** *p* < 0.001.

After determining that short-term exposure to LPS can increase liver size, we next sought to evaluate the long-term effects of LPS exposure. For this, five-month-old adult *kras*+ transgenic zebrafish and their non-transgenic wild-type sibling zebrafish were treated with 10 μg/mL Dox alone or with 10 μg/mL Dox + 40 ng/mL LPS. Wild-type (*kras*−) adult zebrafish treated with 10 μg/mL Dox without exposure to LPS served as controls. The tumor status of all zebrafish in each group was examined at 2 wpi. H&E staining revealed that, following exposure to LPS, *kras*+/LPS zebrafish exhibited enlarged abdomens and obvious signs of liver overgrowth compared with *kras*+ and wild-type (*kras*−) control zebrafish (Figure 6A). H&E staining also showed that liver tumors in both *kras+* and *kras*+/LPS zebrafish ranged from normal liver morphology to HCC and included the following classes: normal, HP, HCA, and HCC (Figure 6B). The classification of liver neoplasm types was based on established criteria as previously studied [41,42,43,44]. A significant increase in mortality was also observed in *kras*+ and *kras*+/LPS transgenic zebrafish compared with wild-type (*kras*−) control zebrafish (Figure 6C). Histological analysis of *kras+* zebrafish revealed normal, HP, HCA, and HCC (2 wpi: 4/20; 3/20; 3/20; 10/20, respectively), whereas *kras*+/LPS zebrafish presented more severe evidence of HCC (2 wpi: 15/15). All wild-type (*kras*−) control zebrafish exhibited normal liver morphology (2 wpi: 20/20) (Figure 6D).

### 3.6. LPS Exposure Exacerbated Liver Tumor Metastasis as Well as Hepatocyte-Specific Expression of twist1a and kras in Double Transgenic Zebrafish

**Figure 7:**
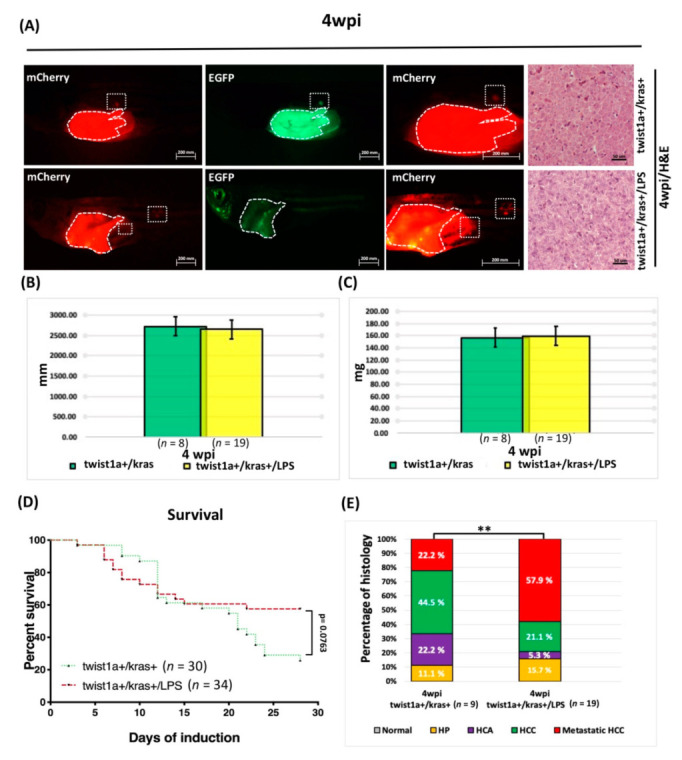
LPS enhanced HCC progression in adult *twist1a*+/*kras*+ transgenic zebrafish. *Twist1a*+/*kras*+ transgenic zebrafish were treated at 3 mpf with Dox and 4-OHT or with Dox, 4-OHT and LPS exposure. Samples were taken at 4 wpi. (**A**) Immunofluorescence analysis of mCherry and EGFP-labeled metastatic liver tumors and H&E staining of liver tissues from *twist1a*+/*kras*+ and *twist1a*+/*kras*+/LPS zebrafish at 4 wpi (white dotted frame: primary and metastatic liver tumors). (**B**,**C**) No significant differences in body lengths or weights were found between *twist1a+*/*kras+* and *twist1a+*/*kras+*/LPS transgenic zebrafish at 2 and 4 wpi. (**D**) Kaplan-Meier survival curves show the percentage of survival up to 4 wpi. (**E**) Histological analysis revealed that *twist1a*+/*kras*+ and *twist1a*+/*kras*+/LPS transgenic zebrafish developed HCC or metastatic HCC at 4 wpi. Scale bar: 50 or 200 μm. Student’s *t*-test or one-way ANOVA were used to assess differences between variables: ** *p* < 0.01.

After our aforementioned research results demonstrated that induction of *kras*+ combined with LPS exposure increased liver size and the incidence of liver tumors, we next investigated how LPS exposure affected liver tumor metastasis in *twist1a*+/*kras*+ double transgenic, adult-stage zebrafish. For this, three-month-old *twist1a*+/*kras*+ zebrafish were treated with 10 μg/mL Dox and 1 μg/mL 4-OHT and exposed to 40 ng/mL LPS. Long-term treatment samples were collected and investigated at 4 weeks (Supplementary Figure S1B). HCC and metastatic HCC were examined using H&E or immunofluorescence analysis (Figure 7A,B). No significant differences in body lengths or body weights were found between *twist1a*+/*kras*+ and *twist1a*+/*kras*+/LPS groups (Figure 7B,C), nor were there any differences in terms of mortality at *twist1a*+/*kras*+ and *twist1a*+/*kras*+/LPS groups (Figure 7D). Histological analysis of *twist1a*+/*kras*+ zebrafish revealed the presence of HP, HCA, HCC, and metastatic HCC in some fish (4 wpi: 1/9; 2/9; 4/9; 2/9, respectively), whereas the *twist1a*+/*kras*+/LPS zebrafish presented more severe metastatic HCC (4 wpi: 3/19; 1/19; 4/19; 11/19, respectively). Thus, the metastatic HCC status of *twist1a*+/*kras*+/LPS transgenic zebrafish was more severe following long-term LPS exposure (Figure 7E).

## 4. Discussion

Recent evidence points to an increasing incidence of HCC among men in countries such as Japan, Italy, France, Switzerland, the United Kingdom, and the United States [46,47,48]. The process from clinical diagnosis to treatment primarily involves medical imaging, surgery, regional tumor treatment, and biotherapy. Although medical research has progressed remarkably with regard to HCC, effective treatments for patients are still lacking. Due to tumor recurrence and metastasis, the relative 5-year survival rate of liver cancer patients remains low [47,48], highlighting that further investigations into the mechanisms governing liver tumor metastasis should remain a top priority.

*TWIST1* is involved in biological processes required for normal growth and development and regulates the expression of many specific genes [49]. However, *TWIST1* has known roles in the carcinogenesis of tumor cells. For example, *TWIST1* plays an important role in the vascular invasion and lung metastasis of tumor cells [11]. Primary tumor cells undergo EMT and, in the process of tumor metastasis, travel through the circulatory system to distant organs. The occurrence of EMT in tumor cells and the stimulation of tumor metastasis are promoted by *TWIST1*. In addition, *TWIST1* inhibits apoptosis and senescence and promotes the immortalization of cells [50]. However, the mechanisms by which *TWIST1* affect the metastasis of tumor cells remains unclear, although mechanisms are believed to differ depending on tumor type. One previous study that employed a *Kras*-induced lung cancer transgenic mouse model found that *Twist1* inhibited cell senescence, thereby accelerating and maintaining the tumorigenic effects of mutant *Kras genes* [51]. In the present study, our results showed that *twist1a*+/*kras*+ double transgenic zebrafish developed spontaneous metastatic tumour. The mortality of *twist1a*+/*kras*+ zebrafish was also significantly higher compared to that of *kras*+ zebrafish (Figure 1) despite the significant increase in liver cell apoptosis in *twist1a*+/*kras*+ zebrafish. However, the percentage of apoptotic liver cells was exacerbated in *twist1a*+/*kras*+ zebrafish compared with *kras*+ zebrafish (Figure 3). These results are also consistent with the previous results of *twist1a*+/*xmrk*+ zebrafish compared with *xmrk*+ zebrafish [33]. Thus, the increase in apoptosis of *twist1a* during the liver tumor metastasis at *kras*+ or *xmrk*+ zebrafish is different from the decrease in apoptosis found in rhabdomyosarcoma [52], which means that it has multiple functions. These observations suggest that cell apoptosis or cell proliferation were key factors in the tumorigenesis or metastasis of liver tumors in *kras*+ or *twist1a*+/*kras*+ zebrafish, which is consistent with findings from our previous study on *twist1a*+/*xmrk*+ transgenic zebrafish [33].

*TWIST1* acts as an EMT regulator and promotes tumor progression through distinct mechanisms. The upregulation of *TWIST1* in HCC cell lines promotes cell proliferation and migration [12]. *TWIST1* also regulates downstream genes such as *E-cadherin* and *vimentin* to promote EMT [53], wherein *E-cadherin* is the first confirmed gene target of *TWIST1*expression. *TWIST1* inhibits *E-cadherin* expression by binding to the E-cadherin promoter, resulting in the downregulation of *E-cadherin* and consequent attenuation of cell-cell adhesion as well as the enhancement of cell migration and invasion. An increase in angiogenesis related to tumor progression is also promoted by *TWIST1*, which acts by increasing the production of vascular endothelial growth factors [54]. Moreover, knockout of *TWIST1* has been found to significantly reduce the number of Vimentin-positive breast tumor cells, which indicates that *Twist1* expression is positively associated with *Vimentin* expression. In specific mouse models, *TWIST1* has also been shown to promote EMT in HCC by regulating vimentin via cullin2 circular RNA [12,55]. The results indicate that E-cadherin and Vimentin proteins are significantly increased or decreased, respectively, in *kras*+ zebrafish compared with *twist1a*+/*kras*+ zebrafish (Figure 4), which is consistent with findings from our previous studies [33,40].

The tumor microenvironment, which is composed of stromal cells, endothelial cells, immune cells, inflammatory cells, cytokines, and extracellular matrix, plays a major role in the initiation and development of HCC [56]. LPS induces inflammation in zebrafish by activating (1) TLR4/myeloid differentiation primary response 88 (MyD88)/nuclear factor kappa-light-chain-enhancer of activated B cells (NF-κB) as well as (2) TLR4/MyD88/mitogen-activated protein kinases (MAPKs) signaling pathways [57]. LPS is a ligand for TLR4 and functions by mediating specific effects of bacterial products. TLR4 is also expressed in different types of cells in the liver, including tumor, hepatic stellate, and kupffer cells [58,59,60]. In a mouse model of HCC, LPS was found to promote angiogenesis by stimulating the activation of hepatic stellate cells via the TLR4 pathway [27]. LPS has also been found to be related to the up-regulation of matrix metalloproteinases (MMPs) expression and activation by other pro-inflammatory signals, and zebrafish is a very suitable in vivo model for studying the regulation and activation of MMPs [61,62,63]. The upregulation of vascular endothelial growth factor (*VEGF*) expression by LPS has also been shown to induce angiogenesis in HCC cells through a STAT3-dependent pathway both in vitro and in vivo [25]. Another previous study found that LPS induced hepatic stellate cells to secrete a variety of pro-angiogenic factors, including *VEGF*, platelet-derived growth factor (*PDGF*), and angiopoietin-1 (*Ang-1*) [27]. Our results, obtained after exposing zebrafish larvae (Figure 5) and adult-stage zebrafish (Figure 6) to LPS, are consistent with those of previous studies that employed zebrafish models of *kras*-induced liver or gut tumors (i.e., these studies also reported that LPS treatment accelerated tumor progression) [32,33,36,64]. In addition, co-expression of *twist1a*+/*kras*+ in zebrafish that had been exposed to LPS was found to exacerbate metastasis compared with *twist1a*+/*kras*+ zebrafish that had not been exposed to LPS, indicating that LPS could activate liver tumor progression and metastasis in *kras* mutants in vivo by cooperating with the *twist1a* gene. On the other hand, we also noticed that the *twist1a*+/*kras*+ group has a higher survival tendency than the *twist1a*+/*kras*+/LPS group. However, there is no statistically significant difference. We speculate that expanding the number of zebrafish will reduce this trend (Figure 7). Together, these results reveal the *twist1a*+ and *kras*+ genes have a cooperative relationship in chronic inflammation, which may contribute to interactions within the immune system that exacerbate the development of tumor metastasis.

## 5. Conclusions

In conclusion, our results indicate that *TWIST1* may be an effective target gene in treating HCC metastasis. This is the first in-vivo demonstration that *twist1a* plays an important role in the maintenance and acceleration of liver tumor metastasis in *kras+* adult-stage zebrafish. We also determined that the inflammatory agent LPS plays a significant role in *twist1a*+/*kras*+ double transgenic zebrafish, which could exacerbate HCC metastasis.

## Data Availability

Data are contained within the article.

## Supplementary Materials

The following supporting information can be downloaded at: https://www.mdpi.com/article/10.3390/biomedicines10010095/s1, Figure S1: Experimental design and long-term treatment samples were collected weekly for investigation.
