# Supplementary material for: Effect of Lipopolysaccharides on Liver Tumor Metastasis of twist1a/krasV12 Double Transgenic Zebrafish
Source: Biomedicines. 2022 Jan 2;10(1):95. doi: 10.3390/biomedicines10010095 (PMC8773574; doi:10.3390/biomedicines10010095)
Supplement: Supplementary file 1 [file biomedicines-10-00095-s001.zip › biomedicines-1480571-supplementary.pdf]

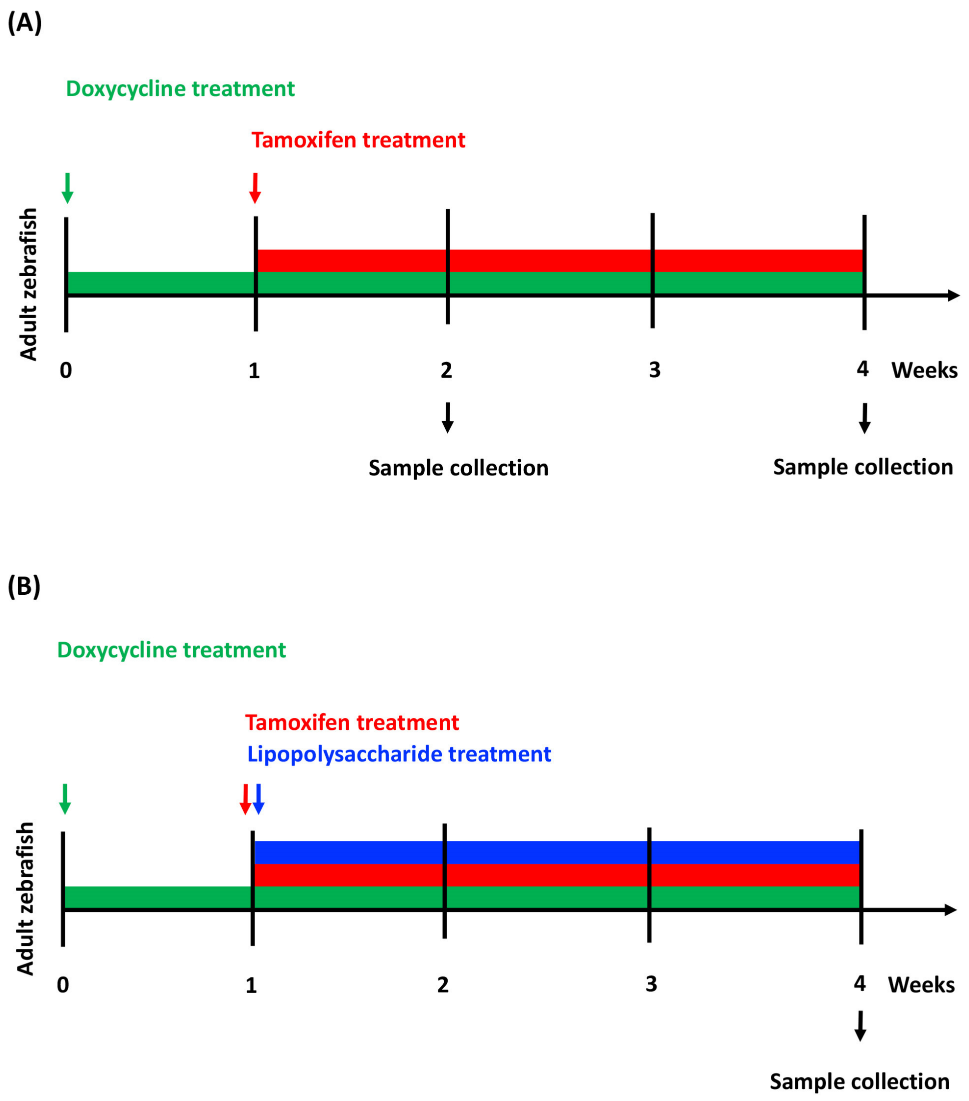

Figure S1. Experimental design and long-term treatment samples were collected weekly for investigation. **(A and B)** A total of different time points were 2 or 2 and 4 wpi.
